# Positive effect of colors and art in patient rooms on patient recovery after total hip or knee arthroplasty: A randomized controlled trial

**DOI:** 10.1007/s00508-021-01936-6

**Published:** 2021-09-07

**Authors:** Sandra Eminovic, Gabor Vincze, Andrea Fink, Stefan F. Fischerauer, Patrick Sadoghi, Andreas Leithner, Lars-Peter Kamolz, Karlheinz Tscheliessnigg, Gerwin A. Bernhardt

**Affiliations:** 1 Department of Orthopedics and Traumatology, Medical University of Graz, Auenbruggerplatz 5, 8036 Graz, Austria; 2 Division of Plastic, Aesthetic and Reconstructive Surgery, Medical University of Graz, Auenbruggerplatz 29, 8036 Graz, Austria; 3 Styrian Hospitals Limited Liability Company, Stiftingtalstraße 4–6, 8010 Graz, Austria

**Keywords:** Quality of life, Effect, Colors, Art, Arthroplasty

## Abstract

**Background:**

Environmental stimuli and well-being are considered to be significant factors in patients’ rehabilitation. The aim of this study was to describe the effect of colors and art in hospital rooms on patients’ recovery after total hip or knee arthroplasty.

**Methods:**

We performed a prospective randomized, controlled study including 80 patients. The intervention group was randomized to colored patient rooms while the control group received medical care in conventional patient rooms. Data were collected preoperatively and postoperatively (3 and 6 days after operation). We measured mood, anxiety and depression, quality of life (QOL) and pain.

**Results:**

Significantly better QOL summary scores were measured in the intervention group (6 days postoperative) compared to the control group (physical component summary score 37.1 ± 5.0 vs. 34.1 ± 6.7; *p* = 0.029 and mental component summary score 51.6 ± 6.6 vs. 47.2 ± 8.4; *p* = 0.015). Postoperatively, we found decreased total mood scores in both groups showing better results for the intervention group without significant differences (*p* = 0.353; *p* = 0.711).

**Conclusion:**

The use of colors in hospital rooms is an effective intervention to improve well-being and to enhance faster rehabilitation. We could demonstrate a positive effect of colors on patients’ postoperative QOL.

## Introduction

Osteoarthritis is one of the most common joint disorders in adults. Due to the demographic change, an increasing number of older people with joint problems can be expected. Affected persons suffer from chronic pain, limited mobility and decreased quality of life (QOL) [1, 2]. The most affected joints are hip and knee. In advanced osteoarthritis total hip or knee arthroplasty is commonly performed, followed by postoperative rehabilitation, to improve the physical function [3]. So far research in total joint replacement has focused on clinical outcomes such as postoperative complications or revisions [4, 5].

Although hospitalization represents a stressful event, the well-being of patients during hospital stay has not been the focus of research to this point. Some studies demonstrated the well-being as a significant factor in patients’ rehabilitation [6–9] and so clinical environment seems to have a significant negative impact on anxiety, stress response, sleep [8–12] and pain tolerance [13]. Frequent interruptions, noise or bright light were described as environmental issues affecting psychological components [14].

The environmental stimuli were found to be determining factors for patients’ behavior [15, 16] and mood [17, 18]. Publications focused on the effects of environment (e.g., on mood or anxiety) in general; however, evidence regarding the impact of colors on patients’ recovery is rare [6]. The aim of this study was to describe the effect of colors in patient rooms on (1) mood, (2) anxiety and depression (3) QOL and (4) pain in patients undergoing total arthroplasty. Our hypothesis was that patients receiving medical care in conventional patient rooms would have inferior scores in outcome parameters.

## Material and methods

We performed a prospective randomized, controlled study including 80 patients. The participants were recruited at a department of orthopedic and trauma surgery at a university hospital. We included patients aged 50 years or older who underwent elective primary total hip (THA) or total knee arthroplasty (TKA). We selected patients planned for elective THA (*n* = 43) or TKA (*n* = 37) because of a comparable length of stay at our department. Intensive care patients were excluded.

**Fig. 1:**
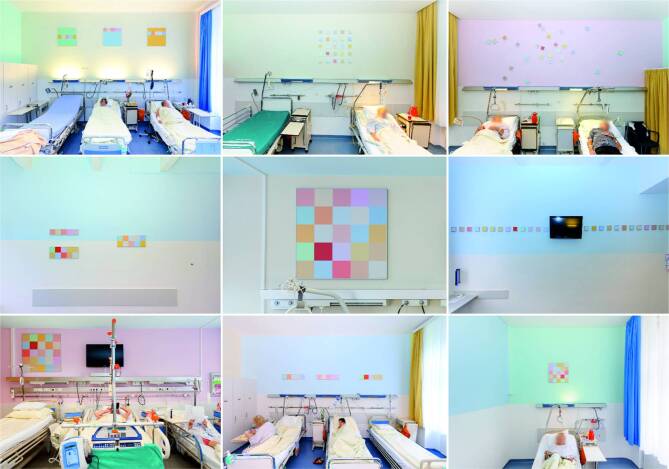
Examples of patients rooms in the intervention group

The intervention group received usual medical care in colored patient rooms. The control group was randomly selected to a conventional patient room with white-colored walls receiving the same medical care. The colors used in the intervention group rooms were selected according to the ideas of an art theorist who developed six color codes for the intervention rooms assuming that light colors have calming effects on mood (Fig. 1). These color codes were additionally framed as pictures (art) and were placed in these rooms (Fig. 1). Each patient was placed in multiperson rooms. Every room included four beds and two windows. Included patients shared the room with three other orthopedic patients over the length of stay. Because the view from the window has an effect on patients’ responses [19], we used rooms with the same outlook in each group.

Patients were evaluated preoperatively (baseline), 3 days after operation (time point 1) and 6 days after operation (time point 2). At each time point we assessed mood, anxiety and depression, quality of life and pain.

Patients’ chronic medical conditions were assessed using the Charlson comorbidity index (CCI) [20] at admission. The mini-mental state examination (MMSE) [21] measured patients’ cognitive state on admission day. Mood was evaluated using profile of mood states brief form (POMS Brief) [22]. The German version of the questionnaire consisted of the four scales: (1) anger, (2) fatigue, (3) vigor and (4) depression. The effect of colours on the anxiety and depression level was evaluated using the hospital anxiety and depression scale (HADS) [23]. Patients answered how they had been feeling over the last 3 days. The evaluation was conducted on admission (as baseline) and 3 and 6 days after the operation. The HADS is a 14-item questionnaire measuring the anxiety and depression level, 7 items measured depression and the other 7 items measured anxiety. Total score ranges from 0 to 21 and each item is rated on a 4-point scale (0–3 points). For QOL assessment, we used the short form health survey (SF-12) questionnaire [24]. The SF-12 as a shorter version of the SF-36 using 12 questions to measure functional health and patient’s well-being. Results are expressed as summary scores, (1) physical component summary (PCS) and (2) mental component summary (MCS). The PCS and MCS scores have a range of 0 to 100. Higher numbers indicate better results. The degree to which pain is related to the environment was estimated by the brief pain inventory (BPI) [25]. We used the validated German version including the key components sensory pain and pain-related impairment.

Questionnaires were handed out to the patients. They received help from a study nurse if necessary in filling out, who was not involved in the study and therefore also blinded to the intervention so that she could not have any impact on the answers of the patients. At the department three medical doctors who performed the study were informed about the intervention; however, neither the surgeons, nurses nor physiotherapists were involved in the study.

For statistical analysis, continuous variables are presented as mean and standard deviation or median and minimum, maximum, and categorical variables are presented as frequencies and percentages. Group comparisons were performed by using *T*-test or Mann-Whitney *U*-test, as appropriate. The statistical analysis was performed using the statistical software SPSS, Version 22 (IBM SPSS Statistics for Windows, IBM Corp, Armonk, NY, USA). *P*-values <0.05 were considered significant. The study was approved by the local institutional review board (number: 27-138 ex 14/15). The work has been reported in line with consolidated standards of reporting trials (CONSORT) guidelines.

## Results

The majority of patients were male (*n* = 45, 56.3%) and the mean age was 67.3 ± 9.4 years. The median CCI was 1.0 (range 0–5) and the median MMSE was 28.0 (range 17–30). Postoperatively, no complications were observed in both groups. The length of hospital stay did not differ between the groups (*p* = 0.864). Demographic data and patient characteristics are presented in Table 1.

**Table 1:** Demographic data and patient characteristics

	Control group *n* = 40	Intervention group *n* = 40	*p*-value	Overall *n* = 80
Gender (female)	20 (20)	15 (25)	0.260	45 (35)
Age (years)	66.8 (± 8.7)	67.7 (± 10.0)	0.687	67.3 (± 9.4)
CCI	1.0 (0–5)	1.0 (0–4)	0.727	1.0 (0–5)
MMSE	28 (17–30)	28.5 (22–30)	0.170	28.0 (17–30)
Length of hospital stay (days)	8.3 (± 2.1)	8.2 (± 1.7)	0.864	8.3 (± 1.9)

Data presented as median and range

### Quality of life

The PCS and MCS scores of the SF-12 increased in both groups continuously after operation. At time point 2 after the operation significantly higher PCS (*p* = 0.029) and MCS scores (*p* = 0.015) were measured in the intervention group. Results of the SF-12 are demonstrated in Table 2.

**Table 2:** Preoperative and postoperative results of the SF-12 and POMS

Outcome	Control group *n* = 40	Intervention group *n* = 40	*p*-value
**Preoperative**			
*SF-12*			
PCS^a^	31.3 (± 6.3)	31.4 (± 6.5)	0.958
MCS^a^	45.4 (± 9.3)	47.5 (± 8.7)	0.328
*POMS*			
Total	85.5 (37–164)	76.0 (35–180)	0.528
Anger	3.0 (0–19)	2.0 (0–21)	0.858
Fatigue	12.0 (0–32)	12.0 (0–31)	0.768
Vigor^a^	16.0 (± 7.7)	15.9 (± 5.7)	0.149
Depression	6.0 (0–52)	8.0 (0–36)	0.724
**Time point 1**			
*SF-12*			
PCS^a^	32.2 (± 5.5)	33.7 (± 6.5)	0.246
MCS^a^	46.1 (± 9.8)	49.5 (± 8.4)	0.094
*POMS*			
Total	34.0 (10–103)	30.0 (5–78)	0.353
Anger	2.5 (0–18)	1.0 (0–16)	0.328
Fatigue	8.0 (0–26)	9.5 (0–24)	0.619
Vigor^a^	15.5 (± 6.8)	15.9 (± 7.0)	0.846
Depression	6.0 (0–56)	5.0 (0–29)	0.521
**Time point 2**			
*SF-12*			
PCS^a^	34.1 (± 6.7)	37.1 (± 5.0)	0.029
MCS^a^	47.2 (± 8.4)	51.6 (± 6.6)	0.015
*POMS*			
Total	33.0 (11–107)	31.5 (14–91)	0.711
Anger	3.0 (0–15)	2.0 (0–15)	0.330
Fatigue	7.5 (0–29)	8.0 (1–27)	0.412
Vigor^a^	15.2 (± 6.4)	16.4 (± 7.0)	0.435
Depression	5.0 (0–54)	4.5 (0–43)	0.794

Data presented as median and range except ^a^mean and standard deviation (SD)

### Mood

Low values of total POMS score were observed in intervention group on time point 1 (median = 30) and time point 2 (median = 31.5) without significant differences (*p* = 0.353; *p* = 0.711). Results of the POMS score are presented in Table 2.

### Anxiety and depression

The mean anxiety and depression score decreased in both groups at time point 1 and 2 after operation without significant difference. Results of the HADS score are demonstrated in Table 3.

**Table 3:** Preoperative and postoperative results of the HADS score and brief pain inventory

Outcome	Control group *n* = 40	Intervention group *n* = 40	*p*-value
**Preoperative**			
*HADS*			
Anxiety	3.0 (0–14)	2.0 (0–12)	0.204
Depression	6.0 (0–14)	3.0 (0–15)	0.067
*Brief pain inventory*			
Sensory pain^a^	16.8 (± 6.6)	16.5 (± 5.1)	0.063
Pain-related impairment^a^	29.7 (± 11.1)	25.9 (± 8.4)	0.300
**Time point 1**			
*HADS*			
Anxiety	3.0 (0–12)	1.0 (0–9)	0.209
Depression^a^	4.3 (± 2.9)	3.8 (± 3.9)	0.769
*Brief pain inventory*			
Sensory pain^a^	17.4 (± 6.2)	16.8 (± 6.3)	0.649
Pain-related impairment^a^	26.9 (± 9.8)	23.6 (± 9.8)	0.162
**Time point 2**			
*HADS*			
Anxiety	3.0 (0–12)	1.0 (0–11)	0.235
Depression^a^	4.0 (± 3.5)	3.2 (± 3.4)	0.462
*Brief pain inventory*			
Sensory pain^a^	16.7 (± 5.2)	14.5 (± 4.3)	0.137
Pain-related impairment^a^	25.7 (± 6.8)	22.8 (± 10.0)	0.119

Data presented as median and range except ^a^mean and standard deviation (SD)

### Pain

The mean sensory pain score of patients remained high in both groups after operation. No significant differences were found in sensory pain at time point 1 (*p* = 0.649) and time point 2 (*p* = 0.137) after the operation in both groups. Pain-related impairment scores decreased on time point 1 after operation (*p* = 0.162) in the intervention (23.6 ± 9.8) and control groups (26.9 ± 9.8). Overall results of the BPI questionnaire are described in Table 3.

## Discussion

To the best of our knowledge this is the first prospective study evaluating the effects of colors and art in patient rooms on the well-being of surgical patients. The aim of this study was to describe the effect of colors and art in patient rooms on patients’ well-being including (1) mood, (2) anxiety and depression (3) quality of life and (4) pain. The hypothesis of the study was that patients receiving medical care in conventional patient rooms would have inferior outcome scores postoperatively. Furthermore, we decided to make measurements on repeated time points to indicate the progress of each outcome during hospitalization. Patients were evaluated preoperatively (baseline), 3 days after operation (time point 1) and 6 days after operation (time point 2). Compared to time point 1 and 2 our results showed worse mood and anxiety scores on admission day in both groups. We assume that this result reflects preoperative stress due to the planned surgical intervention. Previous research showing similar levels of preoperative anxiety [26, 27] confirm our results. Postoperatively, we did not find any significant differences in mood scores or the subscales between groups and the remaining scores (e.g. HADS or BPI questionnaire) did not show significant differences after the operation between the groups except the SF-12 results. We measured higher PCS (37.1 ± 5; *p* = 0.029) and MCS scores (51.6 ± 6.6; *p* = 0.015) on time point 2 in the intervention group. Thus, the results indicate that colored patient rooms have an influence on the postoperative well-being of patients. Comparable results were not available as there is a lack of studies evaluating the same outcomes.

A lack of this study was that we did not evaluate patients’ perceptions of physical conditions in the healthcare setting. Therefore, we could not identify the environmental sources of satisfaction or the overall satisfaction with the hospital experience. When patients’ perceptions are taken into account to predict the satisfaction, they often appear as a significant contributor [28]. Moreover, high-quality research evaluating the impact of environment on patients’ well-being is very challenging because of several influencing factors in hospitals such as light or sound [12]. We used recommended and validated scores [22–25] assessing the described outcomes. It could also have a positive psychological side effect that patient’s rooms were more individualized than normally and patients feel themselves more comfortable and personalized in that environment. Only German versions of scores were applied to interview study patients. We included scores with high results in validity, reliability, sensitivity and specificity [22–25].

Past research analyzed the stimuli of patients to various colors but without conducting the study in a real-life setting [7, 29, 30]. While some other investigations focused on the effects of healthcare environment, in general evidence regarding the impact of colors on patients’ postoperative rehabilitation is lacking [6, 18]. Based on these facts we designed a study in a real-life setting to describe the well-being of surgical patients. We tried to control several influencing factors, e.g. the view from the window or length of stay. Patients were assigned to fully occupied rooms. Our findings offer useful aspects for planning future hospital redevelopments. Based on our results a larger cohort has to focus on effects of healthcare environment including the impact of colors on well-being and rehabilitation. Larger effects were observed at the later time point, following the assumption that more significant results could be demonstrated for prolonged hospitalization time. Studies should therefore focus on patients over longer hospitalization periods, such as in rehabilitation hospitals. Research should examine the environmental effects especially on surgical patients because the preoperative emotional status of patients alters the endocrine and metabolic function [31].

## Conclusion

We could show a significant positive effect of colors on patients’ postoperative QOL. The use of colors in hospital rooms is an effective and low-cost intervention to improve well-being and possibly to enhance a faster rehabilitation. Further studies are needed to confirm our results in a larger cohort. Investigations have to focus on the impact of healthcare environment including other influencing aspects such as light, sound, temperature or patients’ perceptions of the physical environment as previously described [28, 32–35].
